# Relationships of frequencies of extreme low temperatures with grain yield of some Australian commercial chickpea cultivars

**DOI:** 10.1007/s00484-022-02344-9

**Published:** 2022-09-07

**Authors:** Yashvir S. Chauhan, Sam Allard, Steve Krosch, Merrill Ryan, R. C. N. Rachaputi

**Affiliations:** 1 Department of Agriculture and Fisheries (DAF), Kingaroy, Qld 4610 Australia; 2 DAF, Hermitage Research Facility, Warwick, Qld 4370 Australia; 3 Queensland Alliance for Agriculture and Food Innovation, The University of Queensland, Gatton Campus, Gatton, Qld 4343 Australia

**Keywords:** *Cicer arietinum L*., Critical period, Frost, Flowering, Low temperature, Photothermal quotient, Sensitive stage, Yield loss

## Abstract

In this study, we examined the relationships between extremes of low temperatures and chickpea yield in 12 field experiments conducted at six sites in the subtropical environment of southeast Queensland (SEQ) from 2014 to 2019. Three commercial chickpea cultivars, PBA-Boundary, PBA-HatTrick and PBA-Seamer, were grown in all the experiments. Cultivars PBA-Pistol, PBA-Monarch and Kyabra were also included in three of these experiments conducted in 2015. In these experiments, the crop experienced a total of 8 to 41 frosts (minimum temperature <  = 0 °C), 2 to 41 pre-flowering frosts, 2 to 19 frosts during the critical period, 0 to 13 frosts and 2 to 71 low-temperature days (< = 15 °C) after flowering. The mean yield, which varied from 1 to 3 t/ha, was negatively related to post-flowering frosts (*r* =  − 0.74, *p* < 0.01) and low-temperature days (*r* =  − 0.76, *p* < 0.01), and positively related to pre-flowering frosts (*r* = 0.67, *p* < 0.05). Each post-flowering frost was associated with a 5% decrease and a low-temperature day with a 1% decrease in yield. The cultivar × site interaction was significant only in the three experiments with six commercial cultivars. This interaction was most likely due to an increase in the sensitivity range with additional cultivars, as indicated by frost damage scores and their relationships with yield. The results imply that extreme low-temperature events after flowering could negatively impact chickpea yield in SEQ and similar subtropical environments. Overcoming these effects through management and breeding should increase and stabilise chickpea yield.

## Introduction

Chickpea is one of the major pulse crops that can sustainably meet the protein needs of the growing world population (Pyett et al. 2019). Australia, the top exporter of chickpea (Muehlbauer and Sarker 2017), can effectively contribute to this goal by increasing and stabilising production in its broadacre cropping systems while delivering sustainability benefits to its farming systems (Stagnari et al. 2017). In this pursuit of maximising these multiple benefits, chickpea has already become one of Australia’s major broadacre legume crops, with over 1 million hectares of plantings in 2016. In 2018, the area planted to chickpea in Australia was 303,000 ha, and in 2019, 370,000 ha, with an average yield of < 1 t/ha (ABARES 2019). However, the current low yields of this crop will need to be increased to strengthen the crop’s competitiveness in farming systems and retain such a high adoption rate in the future. A significant challenge in achieving this goal could arise from its perceived sensitivity to climatic variability, especially extreme temperature events, including frosts (≤ 0 °C), low (< 15 °C) mean ambient temperatures and heat stress (maximum temperatures > 35 °C). These stresses could exacerbate under climate change (Clarke and Siddique 2004; Upadhyaya et al. 2011). The current improvement efforts on temperature extremes are primarily focused on heat stress, mainly due to the heightened threat of climate change (Jeffrey et al. 2021). However, as a winter crop, chickpea’s vulnerability to low-temperature stress also requires attention (Maqbool et al. 2010; Dreccer et al. 2018; Anwar et al. 2022). Losses in grain yield attributed to extreme low-temperature events, including frosts and low ambient temperatures, are recognised to be a significant problem for chickpea grown in subtropical and temperate environments (Dalal et al. 1998; Croser et al. 2003; Gowda et al. 2009; Chauhan and Ryan 2020; Singh et al. 2016). However, the quantitative relationships of these stresses with grain yield have remained elusive. Also, when the crop is more susceptible to these events has not been well defined.

The literature reviewed suggested that an appreciable reduction in chickpea grain yield can occur when frosts and low temperatures coincide with the reproductive phase (Maqbool et al. 2010; Kaloki et al. 2019). However, there is limited published information to support this view. Oweis et al. (2004) reported that chickpea yields were the lowest in a season with the highest number of frost days. Singh et al. (2016) suspected that the nearly two-fold difference in chickpea yield over the two seasons they observed was due to differences in the number of frosts and low-temperature days in their experiments. However, the crop stage when these occurred was not well defined. Frosts may also exert some positive influence on grain yield. Dreccer et al. (2018) suggested that in the northern regions comprising parts of New South Wales and Queensland states of Australia, days with < 0 °C minimum ambient temperatures before flowering were associated with higher grain yields. The low-temperature effects could also be due to increases in the photothermal quotient. The photothermal quotient integrates the effects of solar radiation and temperature on crop growth and development (Sadras and Dreccer 2015; Dreccer et al. 2018). Higher PTQ before flowering lowered chickpea yields in Australia’s Western part but increased in other environments (Dreccer et al. 2018). This relationship could also be because low minimum ambient temperatures delay flowering by slowing thermal time accumulation. This would minimise exposure to frosts after flowering and assist in developing better crop canopies. Generally, different crops have a critical period of sensitivity to abiotic stresses (Sandaña and Calderini 2012; Lake and Sadras 2014; Kirkegaard et al. 2018). For chickpea, the critical period was defined as 300°Cd before flowering to 500°Cd after flowering (Lake and Sadras 2014). It is not known if chickpea is indeed sensitive to low-temperature extremes during this period. Greater clarity on the sensitivity of different stages of the crop to these stresses is urgently required to develop robust strategies to minimise losses associated with frosts and low temperatures.

In addition to avoiding extreme low-temperature events during the sensitive stages, it will be prudent to improve the crop’s resilience especially when exposures to such events are unavoidable (Heidarvand et al. 2011; Clarke et al. 2004; Clarke and Siddique 2004; Croser et al. 2003; Chaturvedi et al. 2009). In fact, due to climate change, the frequencies of these events could even increase due to the warming of weather and fewer cloudy days (Crimp et al. 2016). Hence, in some environments, the same crop may be exposed to low and high-temperature stresses within a growing season with preponderance in either or both types (Devasirvatham and Tan 2018; Dreccer et al. 2018; Lake et al. 2016). In southeast Queensland, which contributes to significant chickpea production in Australia, the risk of heat stress is relatively small (Chauhan et al. 2017, 2021).

There may also be a need to separately increase the resilience of the crop to low-temperature stress and frosts if they have distinct effects on the crop (Croser et al. 2003). Frost tolerance is one of the most critical components of winter hardiness of different crops (Longin et al. 2013), and some differences among accessions have been noted (Admas et al. 2021; Pouresmael et al. 2020). Effective screening methods, currently not available, will be required to identify putative tolerant cultivars. Frosts in the field environment are also quite unpredictable, posing additional difficulty in such screenings and are not the only factor affecting grain yield in the growing environment. Due to the random nature of frost events, a more pragmatic approach would be to opportunistically compare frost tolerance of commercially relevant cultivars and recommend the more tolerant ones for cultivation in frost-prone areas. A potential advantage of this approach would be that such cultivars will also have other traits such as high grain yield potential or disease resistance for which they may have been bred initially. These approaches remain to be investigated.

The first objective of this study was to determine the relationship between grain yield of commercially relevant cultivars and frequencies of extreme low temperatures at different stages of crop growth. The second objective was to analyse cultivar × environment interactions related to such extremes as a prelude to screening cultivars tolerant to such extreme temperatures.

## Materials and methods

### Experimental protocol

We collected grain yield, flowering and climatic data for this study from 12 chickpea experiments conducted at six sites, including Dalby (27.17°S and 151.26°E), Jondaryan (27.35°S and 151.57°E), Kingaroy (26.58°S and 151.83°E), Oakey (27.49°S and 151.75°E), Warra (26.93°S and 150.93°E), and Warwick (28.20°S and 152.10°E) in subtropical southeast Queensland in Australia from 2014 to 2019 (Table 1). These sites represented the Burnett, Eastern Darling Downs and Southern Downs agro-ecological regions for chickpea production with high frequencies of frost events (Chauhan et al. 2017; Chauhan and Ryan 2020). Each experiment was a unique combination of site-year-sowing-date, which constituted an environment. Within each experiment, three to six cultivars were sown in a randomised block design (Table 1). There were three replications in each experiment. Sowing date at each site was based mainly on planting opportunity characterised by 30 mm rain in 3 days and storage of at least 50 mm water in the soil and creating variation in low-temperature extremes; hence, sowing dates varied across the experiments. For logistical reasons, the row spacing was kept at 76 cm at Hermitage and 90 cm at other sites. The slight variation of 14 cm in row spacing across sites was not expected to alter the influence of climatic factors being investigated, especially since the target plant population was standard 30 plants/m^2^ (Felton et al. 1996). Sowing for each experiment was done using a cone planter which placed enough seeds to establish around 30 plants/m^2^. All experiments were raised as rainfed except the experiment sown on 23 April 2015 at Kingaroy, which received one irrigation on 12 August 2015, and the experiment sown at Oakey, which received two irrigations (30 mm) on 17 June and 12 August 2017.

**Table 1:** Site-year-sowing date, soil, plant available water holding capacity (PAWC), and weather descriptors including rain, average maximum (MaxT) and minimum (MinT) temperatures and photothermal quotients (PTQ) before (Prefl.) and after flowering (Postfl.) in 12 field experiments (Exp.). Each site-year-sowing date combination constituted a distinct environment

Exp									PTQ	
(#)	Site	Year	Sowing-date	Soil	PAWC	Rain	MaxT	MinT	Prefl	Postfl
					(mm)	(mm)	°C	°C	MJ/°Cd	
1	Dalby	2014	20-May	Vertosol	285	111	23.1 ± 4.2	6.5 ± 5.1	0.54	0.57
2	Warra	2014	16-May	Ferrosol	208	108	23.3 ± 4.0	6.6 ± 4.9	0.55	0.56
3	Kingaroy^a^	2015	23-Apr	Ferrosol	109	169	21.5 ± 3.5	6.8 ± 4.9	0.44	0.59
4	Jondaryan^a^	2015	24-Apr	Vertisol	150	210	21.5 ± 4.0	6.7 ± 4.6	0.49	0.61
5	Warwick^a^	2015	27-Apr	Vertisol	220	183	20.7 ± 4.4	5.4 ± 4.9	0.57	0.59
6	Kingaroy	2015	13-May	Ferrosol	109	154	22.2 ± 3.7	6.7 ± 5.0	0.47	0.58
7	Kingaroy	2016	4-Apr	Ferrosol	109	128	22.5 ± 3.9	8.0 ± 5.1	0.38	0.44
8	Jondaryan	2016	18-Apr	Vertosol	278	276	21.9 ± 4.4	7.5 ± 4.9	0.38	0.56
9	Warwick	2016	4-Jun	Vertosol	200	409	20.5 ± 4.4	7.4 ± 4.4	0.51	0.61
10	Oakey	2017	17-Jun	Vertosol	200	189	24.1 ± 4.5	7.5 ± 6.2	0.56	0.47
11	Warwick	2017	26-Jun	Vertosol	200	123	23.4 ± 4.5	5.9 ± 7.1	0.44	0.60
12	Kingaroy	2019	May	Ferrosol	109	106	23.3 ± 4.5	6.51 ± 4.7	0.47	0.57

^a^Experiments with six cultivars, including PBA Boundary, PBA HatTrick, PBA Seamer, PBA Pistol, PBA Monarch (Kabuli) and Kyabra. All other experiments included only cultivars PBA Boundary, PBA HatTrick and PBA Seamer. MaxT and MinT are the averages across the growing period ± standard deviation and photothermal quotients were the averages for the pre- (Prefl.) and post-flowering (Postfl.) phases of growth.

### Observations

Time to 50% flowering was recorded when at least 50% of the plants had one open flower and maturity when over > 80% of pods on all plants were ready for harvesting. Thermal time (°Cd) to flowering was computed by summing the daily mean ambient temperatures prevailing between sowing and flowering. The critical period frosts (CPFr) were the frosts that occurred during the critical period, defined by Lake et al. (2016) as the growth period spanning 300-^o^Cd before and 500-^o^Cd after 50% flowering. Days with minimum temperature ≤ 0 °C before 50% flowering were the pre-flowering frosts (PreFFr), and after 50% flowering, post-flowering frosts (PostFFr). The sum of PreFFr and PostFFr was the total number of frosts (TFr). Days with mean ambient temperature being < 15 °C after 50% flowering were the low-temperature days after flowering (LTDF). The extent of frost damage was scored on a 1–9 scale in September, with one as minor visible damage and nine as the worst damage in two experiments conducted at Kingaroy and Jondaryan in 2015. At Hermitage, the frost damage was least apparent. In all experiments at maturity, a hand-harvested sample from representative areas of about 2 m^2^ was taken from each plot to estimate grain yield.

Temperatures, rainfall and incident solar radiation were monitored 1.5 m above the soil near each experiment. The photothermal quotient (PTQ, MJ m^−2^°Cd^−1^) was calculated separately for the before and after flowering periods using the following equation (Fischer 1985):

1 $$\mathrm{PTQ} =\frac{\sum_{\mathrm{start}}^{\mathrm{end}}\mathrm{PAR}}{\overline{T}\mathrm{start }-\mathrm{end}}$$

where PAR was photosynthetically active radiation, and *T* was temperature between the ‘start’ and ‘end’ of the before and after flowering periods.

### Statistical analysis and grain yield loss calculations

The coefficient of variation (CV%) in different parameters across the 12 environments was computed by dividing the respective standard deviation by the mean and multiplying the resulting value by 100. For yield, CV% was calculated by dividing the root mean square error by the average yield of only three cultivars, PBA Boundary, PBA HatTrick and PBA Seamer and multiplying by 100.

The data analysis tool in Excel generated a correlation matrix between different plant attributes, including grain yield and flowering time with temperature and frost events, and photothermal quotients during the pre- and post flowering periods. The key relationships of interest, including frosts occurring before and after flowering and mean temperatures < 15 °C were analysed using regression analysis. The grain yield loss per unit increases of the significantly harmful stress factors, including frosts and temperature after flowering being < 15 °C, was computed using the following equation.

2 $$\mathrm{Grain}\;\mathrm{yield}\;\mathrm{loss}\;\mathrm{per}\;\mathrm{event}=\frac{\mathrm{Slope}}{\mathrm{Intercept}}\times100$$

The intercept in Eq. 2 represented the potential grain yield without any constraint. The slope value represented the rate of loss in grain yield. The experiments where yields did not conform to the general trend were also considered to be affected by other factors, and the relationship was analysed with and without including data from these experiments.

Each experiment in Table 1 constituted one of the 12 environments resulting from a different site-year-sowing date combination and represented part of the variability in frequencies of temperature stresses that chickpeas can experience in southeast Queensland and cultivars included in them as treatments. In three experiments in 2015, there were six cultivars, including three common cultivars in all environments. The sowing date of each experiment was primarily selected based on planting opportunity and creating variability in the frequency of frosts and low-temperature days coinciding with different stages of crop growth. The cultivar × environment interactions were analysed using AMMI analysis (Gauch 2013) for all 12 experiments with three common cultivars and separately for three experiments conducted in 2015 with three additional cultivars. Frost damage scores recorded in September and harvest yield in three experiments conducted in 2015 at Jondaryan and Kingaroy established the relationship between frost damage scores and yield. The level of significance was set at 5% for all tests.

## Results

### Weather and frequencies of frosts and low temperatures at different stages

All 12 experiments, which constituted 12 distinct environments, had maximum seasonal average temperatures ranging between 20 and 24 °C, minimum temperatures between 5.4 and 8 °C, and in-season rainfall between 106 and 409 mm (Table 1). The crop only experienced a maximum temperature of > 35 °C for 3 days at Oakey in 2017, 1 day at Warwick in 2017 and 2 days at Kingaroy in 2019 (data not shown). The photothermal quotient during the pre-flowering varied from 0.38 to 0.57 and during the post-flowering periods from 0.44 to 0.61. The crop experienced 8 to 41 TFr, 2 to 19 CPFr, 2 to 41 PreFFr, 0 to 13 PostFFr and 2 to 71 LTDF in different experiments (Table 2). Warwick had the highest number of CPFr in 2015, Tfr and PreFFr in 2017, but PostFFr were the highest at Kingaroy in 2015 and LTDF at the same place in 2016. The coefficient of variation for all the frost events and low-temperature days was > 50%.

**Table 2:** The frequencies of total frosts (TFr), frosts during the critical period (CPFr), pre-flowering frosts (PreFFr), post-flowering frosts (PostFFr) and the number of days with low temperature after flowering (LTDF) during the reproductive phase in 12 experiments conducted at six sites in southeast Queensland from 2014 to 2019

Site-year	TFr	CPFr	PreFFr	PostFFr	LTDF
Dalby14	23	6	17	2	12
Warra14	27	11	22	2	11
Kingaroy15^a^	16	13	3	13	48
Jondaryan15^a^	16	11	11	5	33
Warwick15^a^	32	19	29	3	32
Kingaroy15	19	14	13	6	34
Kingaroy16	9	8	2	7	71
Jondaryan16	14	8	6	8	43
Warwick16	8	8	8	0	25
Oakey17	17	5	16	1	2
Warwick17	41	3	41	0	4
Kingaroy19	14	2	12	2	32
Mean	19.4	9.0	15.0	4.0	31.6
CV%	50.6	54.2	77.2	94.8	60.9

^a^Experiments with six cultivars, including PBA Boundary, PBA HatTrick, PBA Seamer, PBA Pistol, PBA Monarch (Kabuli) and Kyabra. All other experiments included only three cultivars, including PBA Boundary, PBA HatTrick and PBA Seamer.

### Flowering and grain yield

The mean days to flowering ranged from 59 days at Kingaroy in 2016 to 111 days at Warwick in 2015 (Table 3). The thermal time to flowering varied from 1017 to 1435°Cd. As only the environment effect was significant for yield in 12 experiments with three common cultivars, PBA Boundary, PBA HatTrick and PBA Seamer, their environmental mean yields are presented in Table 3. Across these experiments, the average grain yield of the three cultivars ranged from 1.0 to 3.1 t/ha. Grain yields were generally lower at Kingaroy and were less than 1.5 t/ha in three out of four experiments at this site (Table 3). There was slight lodging in 2016, waterlogging, and terminal drought in 2019 at this site. Grain yields were also low at Jondaryan in 2016 when there was some Ascochyta blight. In all other experiments, grain yield averages were > 1.5 t/ha. The CV% for all the three traits was < 16.8%.

**Table 3:** Days to 50% flowering, thermal time to 50% flowering and average grain yield of three cultivars in 12 experiments conducted at six sites in southeast Queensland from 2014 to 2019, and coefficient of variation (CV) across environments

Site-year	50% flowering	Thermal time (TT)	Grain yield
	(d)	(°Cd)	(t/ha)
Dalby14	90	1156	2.73
Warra14	91	1172	2.16
Kingaroy15	73	1132	1.32
Jondaryan15	95	1242	2.14
Warwick15	111	1285	2.81
Kingaroy15^b^	80	1049	2.16
Kingaroy16	59	1079	0.99
Jondaryan16	100	1435	1.03
Warwick16	98	1187	3.09
Oakey17	89	1084	2.47
Warwick17	91	1017	2.95
Kingaroy19	81	1048	1.42
Mean	88	1157	2.11
CV%	15.4	10.3	16.8

^a^Grain yield represents mean of cultivars PBA Boundary, PBA HatTrick and PBA Seamer.

### Relationships of yield and other traits with temperature factors

The mean grain yield in the 12 environments with only three common cultivars was positively correlated to days to 50% flowering (*r* = 0.59, *p* < 0.05) but not to thermal time to flowering (Table 4). The correlation of grain yield was also positive with the number of PreFFr (*r* = 0.67, *p* < 0.05), but negative with PostFFr (*r* =  − 0.74 *p* < 0.01) and LTDF (*r* =  − 0.76, *p* < 0.01). LTDF and PostFFr were also significantly related to each other (*r* = 0.73, *p* < 0.01). The CPFr frosts were not significantly related to grain yield. PTQ-Prfl was also signficantly associated with LTDF. The grain yield relationship was significantly positive with  PTQ-Prfl (*r* = 0.71, *p* < 0.01) but not with  PTQ-Pofl.

**Table 4:** Pearson correlation^a^ coefficients of relationships between different crop and weather attributes

	PAWC^b^	RF	TT	DF	TFr	PreFFr	CPFr	PostFFr	LTDF	PTQ-prfl	PTQ-pofl
RF	0.23										
TT	**0.58**	0.48									
DF	**0.67**	0.43	**0.62**								
TF	0.31	-0.46	-0.14	0.40							
PreFFr	0.33	-0.32	-0.20	0.48	**0.95**						
CPFr	-0.09	0.08	0.39	0.24	0.10	-0.05					
PostFFr	-0.35	-0.08	0.24	-0.45	-0.36	**-0.62**	0.42				
LTDF	-0.42	-0.05	0.22	-0.53	-0.54	**-0.69**	0.35	**0.73**			
PTQ-Prfl	0.32	0.03	0.00	0.55	0.32	0.41	0.24	-0.57	**-0.62**		
PTQ-Pofl	0.12	0.21	0.23	0.55	0.32	0.30	0.25	-0.07	-0.27	0.18	
Yield	0.38	0.22	-0.15	**0.59**	0.52	**0.67**	0.05	**-0.74**	**-0.76**	**0.71**	0.42

^a^The critical values of significant correlation coefficients were 0.576 at *p* <  = 0.05 (bold font), and 0.708 at *p* <  = 0.01. Correlations significant at 5% have the bold font. ^b^Variables: *PAWC*, plant available water holding capacity; *RF*, rainfall; *TT*, thermal time; *DF*, days to flowering; *TFr*, total frosts; *PreFFr*, pre-flowering frosts; *PostFFr*, post-flowering frosts, *CPDFr*, critical period frosts, *LTDF*, low-temperature days after flowering, *PTQ-Prfl*., preflowering photothermal quotient; *PTQ-Pofl*., post-flowering photothermal quotient.

**Fig. 1:**
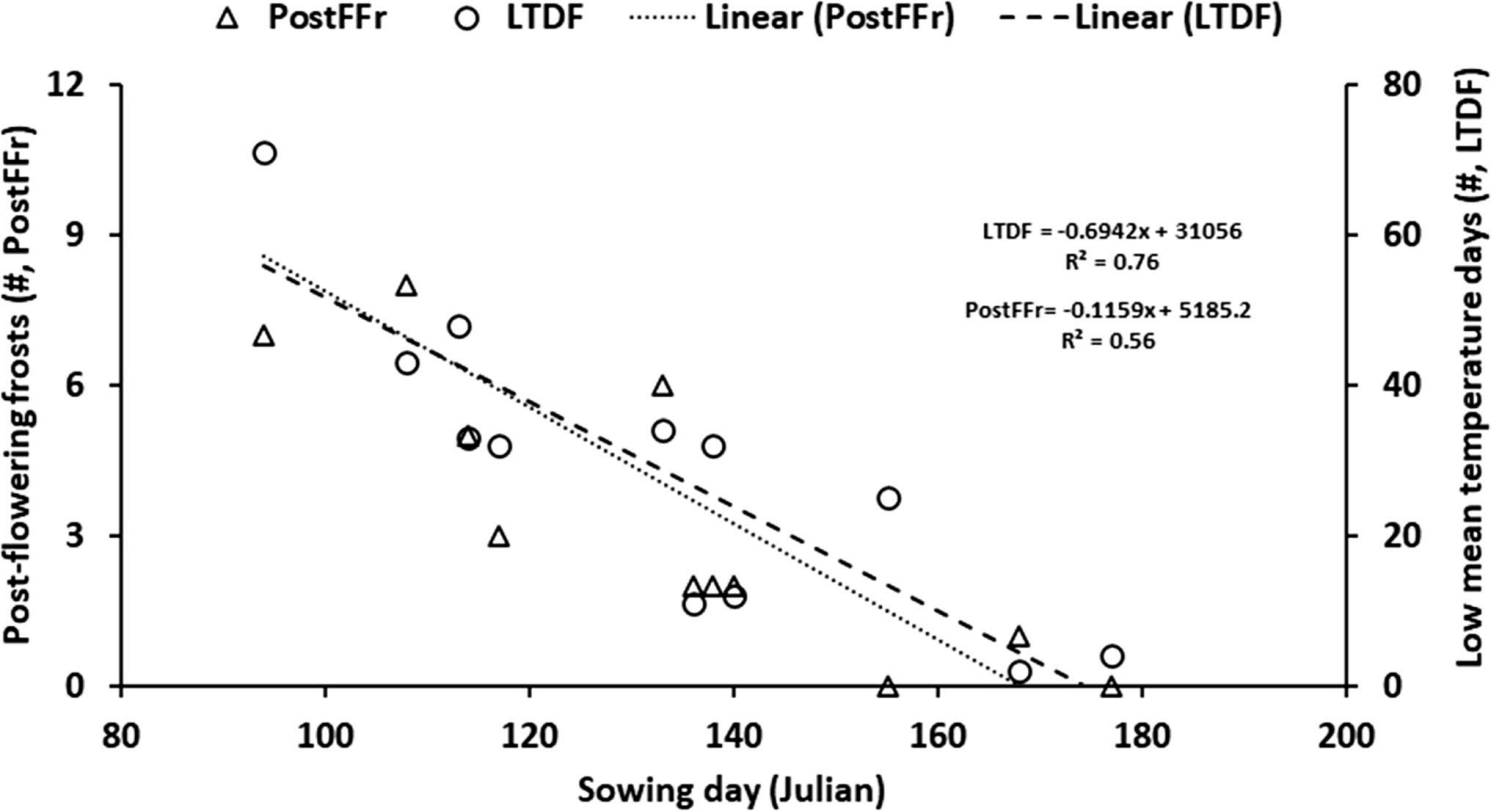
Relationship of date of sowing with frequencies of post-flowering frosts (PostFFr) and low-temperature days (LTDF). The regression, intercept and slopes were significant (*p* =  < 0.01) for both parameters

The frequencies of PostFFr and LTDF were significantly related to the sowing date. Sowing day (Julian) across environments explained 56% variation in the frequencies of PostFFr and 76% variation in the frequencies of LTDF (Fig. 1). This relationship was much stronger than for any other climatic parameter given in Table 2.

**Fig. 2:**
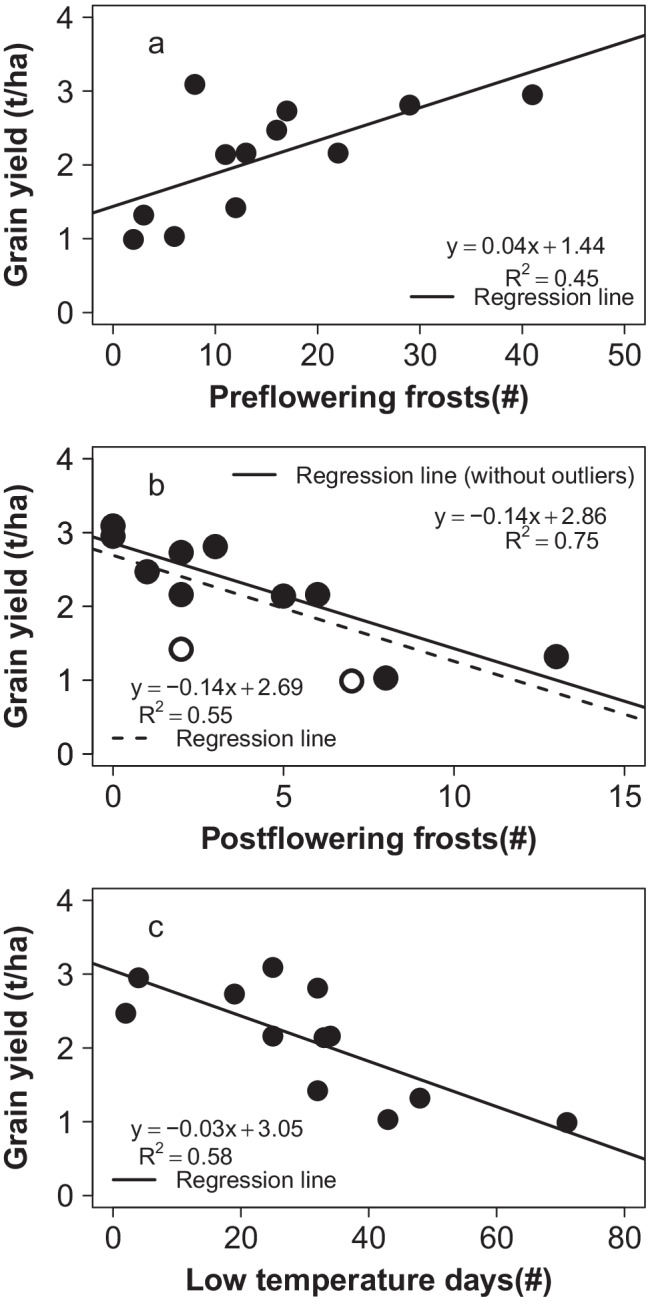
Regression of grain yield with the total number of **a** pre-flowering frosts, **b** post-flowering frosts and **c** low-temperature (< = 15 °C) days. The regression was significant (*p* < 0.05) for ‘a’ and highly significant ( *p* < 0.01) for ‘b’ and ‘c’. The open circles in ‘b’ are possible outliers and the relationship represented solid trend line was drawn ignoring these points. The slopes and intercepts were highly significant (< 0.01) for all the three relationships except for ‘a’ where the intercept was significant only at *p* < 0.05

PreFFr explained about 45.2% of the total variation in grain yield. In contrast, PostFFr explained 54.8% and LTDF 58.4% of the total variation in grain yield (Fig. 2). Two open points in the relationship of PostFFr and yield in Fig. 2 were treated as outliers as they reduced the strength of the relationship between grain yield and PostFFr. When these outliers were ignored, the number of PostFFr explained 74.5% variation in yield. This change, however, did not significantly change the slopes of their relationships and decrease in yield per post-flowering frost. Each PreFFr event was associated with a 2.8% increase in grain yield over 1.47 t/ha. In contrast, each PostFFr and LTDF event was associated with a 5% and 1% loss in grain yield, respectively.

### Cultivar × environment interaction for grain yield

Multi-environment trial analysis with only three cultivars, including PBA Boundary, PBA HatTrick and PBA Seamer, which were included in the 12 environments using the AMMI procedure, revealed significant site differences accounted for 94% of the total variation in sums of squares (Table 5). The cultivar differences and their interaction with the environment, however, were not significant. In three experiments with six cultivars conducted in 2015 at Kingaroy, Warwick and Jondaryan, the cultivar × environment interaction in yield was highly significant (Table 6). The average yields of six cultivars in the three environments were slightly less than the average yields of the three cultivars in the same environments (Table 3).

**Table 5:** Analysis of variance of three and six cultivars. Environments were 12 experiments (Exp) conducted with three cultivars from 2014 to 2019 and three experiments with six cultivars in 2015

Source of variation	Three cultivars		Six cultivars	
	Df	F-ratio	Df	F-ratio
Cultivar (Cv.)	2	1.1	5	41.02***
Rep (environment)	24	1.21	6	18.35***
Environment	11	33.24***	2	26.39**
Cultivar x Env	22	1.21	10	5.70***

* Significant at 5%,** significant at 1%, and *** significant at 0.1% probability.

**Table 6:** Grain yield (t/ha) of six commercial chickpea cultivars at Warwick, Jondaryan and Kingaroy in 2015

Cultivar	Environment		
	Warwick	Jondaryan	Kingaroy
PBA Seamer	2.77	2.27	1.29
Kyabra	2.78	2.52	1.15
PBA Monarch	1.73	1.69	0.81
PBA Boundary	2.96	2.09	1.44
PBA HatTrick	2.69	2.06	1.25
PBA Pistol	2.51	1.76	0.97
Mean	2.57	2.07	1.15
Average LSD	Environment	0.275	
	Cultivar	0.093	
	Cultivar × environment	0.264	

### Relationship of the degree of frost damage scores with yield

**Fig. 3:**
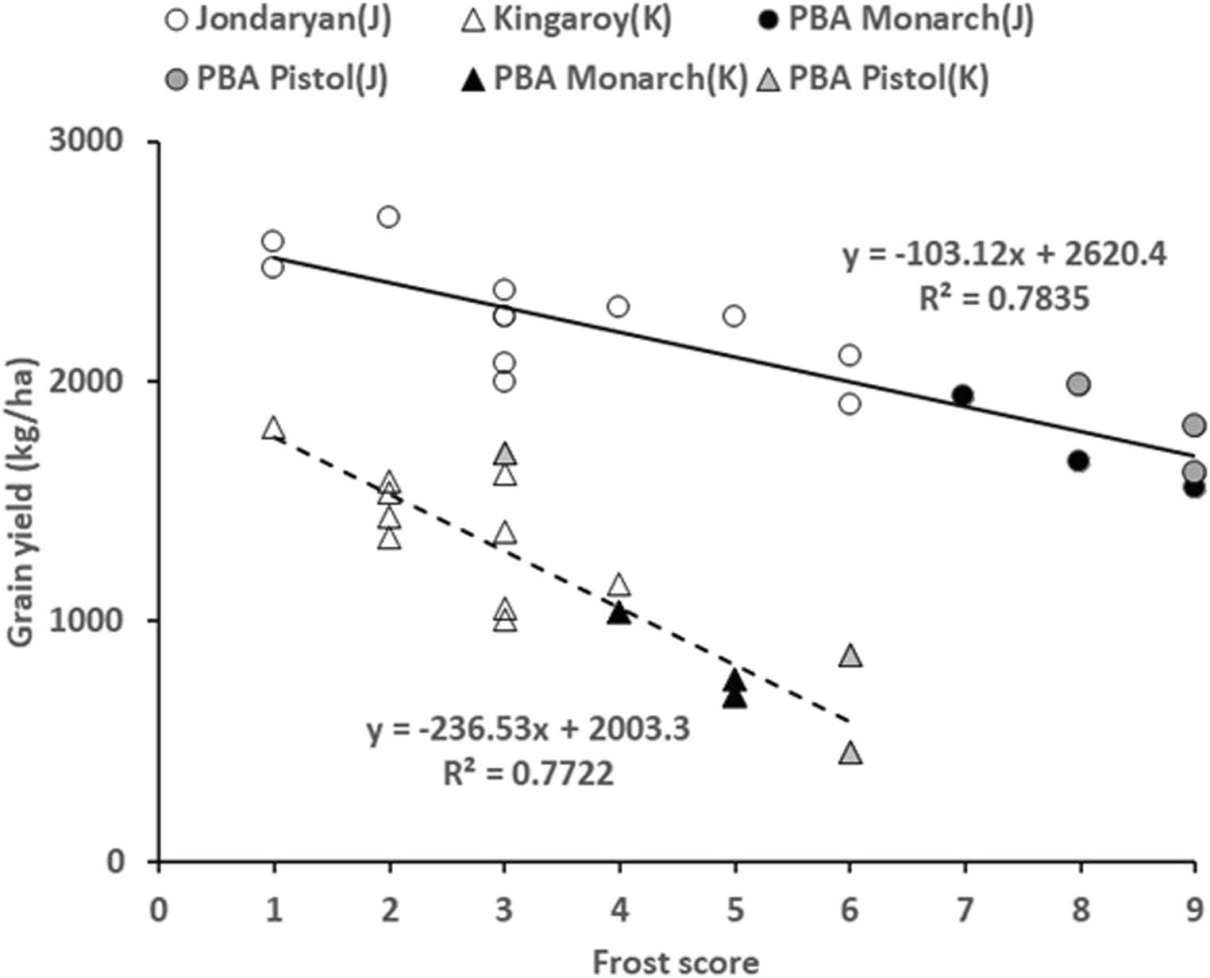
Relationship of frost damage scores of six cultivars and yield in 2015 experiments conducted at Jondaryan and Kingaroy. Each data point represents an individual replication. The frost scores of susceptible cultivars PBA Pistol and PBA Monarch are shaded light or dark

There was visible frost damage at Jondaryan and Kingaroy in 2015. The differences in frost damage scores were smaller, but the slopes of the relationships with yield were significantly different. The slope of the relationship was steeper at Kingaroy than at Jondaryan (Fig. 3). At both sites, frost damage scores accounted for a similar degree (~ 77%) of the total variation in yield. The frost damage scores of PBA Monarch and PBA Pistol were the highest and yielded lowest at both sites (Fig. 3).

## Discussion

Wery et al. (1993) were probably first to categorise three major stress types related to temperature extremes in chickpea. These categories included freezing — when the daily minimum temperature falls < 0 °C, but there was no snow cover, chilling temperatures when the daily average temperature was between 0 and 12 °C. This threshold was increased to 15 °C by Clarke and Siddique (2004), and heat stress when daily maximum temperature was above 25 °C. The threshold for heat stress was increased to 35 °C (Upadhyaya et al. 2011; Devasirvatham and Tan 2018). In this study, our focus was limited to low-temperature extremes as there were only 1 to 3 days when temperatures were > 35 °C and in only three experiments. The effects of high temperature, therefore, were ignored. The frost and low-temperature stresses considered in our study were closer to Wery et al.’s (1993) definition of freezing and chilling temperatures, respectively. Such stresses affect chickpea’s biochemical pathways as defence mechanisms but cannot prevent yield losses (Bhandari et al. 2017). Singh et al. (2016), while studying the effects of irrigation on yield and water productivity, reported around two-fold differences in yield and biomass of chickpea in Ludhiana in India in the 2007/2008 and 2008/2009 winter seasons having similar rainfall. They suspected yield differences in the two seasons could have occurred due to differences in frequencies of extreme low temperatures and frosts in the first 6 to 10 weeks of growth from sowing spanning the vegetative to early flowering stages. Our study provided scope for a more direct assessment of the impact of frequencies of low temperatures on yield due to increased variation in the growing environment created by combinations of six sites, four seasons and different sowing dates.

In our study, we noted up to three-fold differences in the yield of chickpea in the 12 environments. A large proportion of this variability (> 55%) was associated with frosts and low-temperature days after flowering. Losses in yield due to high frequencies of frosts during the reproductive period have also been reported in other crops. In canola, yield losses can be between 2 and 10% for each day of minimum temperatures after flowering or during the critical period being < 0 °C (Lilley et al. 2015; Takashima et al. 2013). In wheat, Zheng et al. (2015) found that a decrease in 10% yield per frost event after anthesis reasonably predicted decreases in wheat yield in the Australian wheat belt. Wang et al. (2015) found that up to 22 to 44% variation in wheat yield was accounted for by the number of frost days (< 2 °C) in different regions of New South Wales. The number of cooler days with < 20 °C was also reported to be related to the tuber and forage yield of potato and alfalfa in Iran (Soureshjani et al. 2019). In chickpeas, Clarke and Siddique (2004) demonstrated that temperatures < 15° C reduced reproductive success through its effects on pollen germination, pollen tube growth and cultivar differences found in this sensitivity. Previous studies on chickpea have indirectly alluded to these temperature effects on yield (Horn et al. 1996; Whish et al. 2007; Singh et al. 2016; Clarke and Siddique 2004) but did not establish their quantitative relationships. This observation, to our knowledge, is the first time that such a direct quantitative impact of frost and low temperatures on grain yield has been shown to occur in chickpea grown in Australia.

Although the extent of yield losses accounted for by frosts appeared to be only about 55%, it could be even more. Two points in the regression that seemed to be outliers in the relationship of PostFFr (Fig. 2b) were related to lodging in 2016 and terminal drought in 2019 at Kingaroy. Chickpea is also highly sensitive to losses in grain yield due to lodging (Raiya et al. 2021) apart from drought (Devasirvatham and Tan 2018; Arif et al. 2021), which could have impacted the relationship of frosts with grain yield. After ignoring these outlier points, the number of post-flowering frosts explained 74% of the variation in grain yield (Fig. 2b). The close relationship suggests that other stress factors, including soil moisture status, may affect the relationship between frost and grain yield. The strong negative relationship of grain yield with the number of frosts occurring after flowering (PostFFr) suggests it may be mainly their number, rather than the intensity on an individual day, that may matter most when accounting for the frost effects. This knowledge may also simplify developing a strategy for managing frost losses in the crop by manipulating crop phenology.

Significant variability (> 65%) in grain yield in our study was also accounted for by LTDF (< = 15 °C). As both low-temperature days and post-flowering frosts were significantly related because they seem to co-occur, separate studies will be required to clarify whether their effects are distinct. Croser et al. (2003) considered them independent abiotic stress factors impacting chickpea yield, while Anwar et al. (2022) clubbed them to analyse their combined effects as the chilling effects. Frost can damage cells and kill the tissue, while low temperatures could reduce pod set by affecting pollen viability and fertilisation. Artificial turbulence of air above the chickpea canopy using fans, which can reduce frosts, could be used to separate temperature effects in future studies. If the impact on chickpea grain yield is primarily due to frost or low temperature, it will be prudent to screen cultivars for both or the most relevant of these two stresses. In our study, frost damage was more conspicuous in two experiments conducted at Jondaryan and Kingaroy in 2015. We found that frost damage scores in these two environments accounted for about 77% of the total variation in grain yield (Fig. 3) but had significantly different slopes. The differences in the slopes of the relationship in the two environments could be due to differences in soil water availability, with Jondaryan soil having higher soil water holding capacity permitting a greater recovery from frost damage. There could be other factors involved too. In South Australia, a report indicated that the degree of frost damage was higher on lighter sandy soil than on darker soil due to differences in soil temperature and moisture (see grdc.com.au/resources-and-publications/grownotes/crop-agronomy/chickpea-southern-region-grownotes/GrowNote-Chickpea-South-14-Environment.pdf).

We found that each post-flowering frost event was associated with a decrease in the grain yield of 5% and LTDF by 1.0%. This estimate of grain yield loss is valuable information for crop modellers as, currently, various chickpea models cannot account for losses in grain yield due to these factors. Because of this, the environments where these temperature-related stresses occur tend to have greater observed and modelled grain yield discrepancies. Correction of simulated yield using these estimates improved grain yield prediction by the APSIM chickpea model (Anwar et al. 2022).

Lake and Sadras (2014) considered improving abiotic stress tolerance in chickpea during the critical period, which they defined as 800°Cd long, with only 2/3rd of this period falling after flowering. Chickpea may not be more vulnerable to frost during the critical period, as our study revealed no relationship between the number of frosts during this period and yield. It could be because, within the critical period, frosts occurring before flowering could have increased grain yield, while frost occurring after flowering reduced grain yield. We obtained one of the highest grain yields (~ 3t/ha) in the experiment conducted at Warwick in 2017 (Table 3), in which the crop experienced up to 41 frost events, but all of them were during the pre-flowering period. Hence, frosts in the early stages may even be beneficial. Most frost management strategies, therefore, aim to limit their occurrences to the pre-flowering period (Anwar et al. 2022; Chauhan and Ryan 2020). A positive association between PreFFr and grain yield alluded to this possibility. Due to low ambient temperatures, pre-flowering frosts may reduce crop water use before flowering by reducing evapotranspiration demand. This possibility was also indicated for chickpea grown in the northern region of Australia by Dreccer et al. (2018). Zaman-Allah et al. (2011) had previously stated that a conservative use of water before flowering was beneficial for grain yield in chickpea. Rachaputi et al. (2021) reported that when the chickpea canopy was artificially reduced during the pre-flowering phase, the crop extracted more water after flowering compared to the control plants resulting in the minimal effect on yield. More studies may be needed to prove that frost and low temperatures before flowering will minimise depletion of soil water reserves which the crop can use later during the flowering and podding periods. Even though the preflowering PTQ was not positively related to PreFFr, it was also positively related to yield. This could be because of cooler temperatures which would be the main factor contributing to a higher PTQ in more productive environments by lengthening the preflowering phase (Dreccer et al. 2018) and eventually reduce the frequency of LTDF as indicated by a significant negative relationship between preflowering PTQ and LTDF (Table 4).

Since the post-flowering stage appears to be the main sensitive stage for frosts and low temperatures that negatively impact grain yield, it could provide a significant opportunity to lessen these impacts by manipulating the flowering time. For this, accurate prediction of flowering time will be required. However, variation in thermal time targets for flowering (Table 3), which are used for predicting flowering time in different environments, could make prediction somewhat tricky. This variation in the thermal time target for flowering seems to be because flowering is modulated by soil water in addition to photoperiod and temperature (Li et al. 2022; Chauhan et al. 2019). The strong negative relationship of PostFFr and LTDF with the day of sowing (Fig. 1) suggests that delaying sowing could also be a more effective means of minimising crop exposure to these stresses to increase yield. However, delayed sowings could increase the risk of terminal drought and heat stress in some environments (Lake et al. 2016; Chauhan et al. 2021), especially in variable and changing climates. As shown by Chauhan et al. (2019), the dynamic modulation of flowering time with soil water complicates the reliance on this strategy (Chauhan and Ryan 2020). Since soil water storage varies from field to field depending upon soil types and rotations followed, there may be a need for a more accurate prediction of flowering time for individual paddocks (Chauhan and Ryan 2020). Chauhan et al. (2019) have modelled the dynamic effect of soil moisture on chickpea flowering, providing a more effective assessment of frost risk in chickpeas under variable soil moisture. This information may help assess the usefulness of agronomic interventions, including sowing times or irrigation that may alter flowering time to reduce the risk of frosts and low temperatures impacting yield while ensuring the severity of terminal drought is minimised.

Another important finding in this study was detecting a highly significant cultivar × site interaction in grain yield, with six cultivars included in three experiments conducted at Warwick, Jondaryan and Kingaroy in 2015. In these experiments, fewer PostFFr frosts and low-temperature days were observed at Warwick, where higher yields were obtained compared to Kingaroy and Jondaryan (Tables 2 and 3). The interaction may have been significant due to differences in individual cultivar response to the environment to which frosts and ambient temperature could have been major contributory factors. The cultivar × environment interaction suggests that cultivar rankings could change if the crop was exposed to frosts during the sensitive period. Cultivar PBA Pistol and Monarch included in these three experiments appeared to be particularly sensitive to post-flowering frosts. This was also revealed by high frost damage scores of these two cultivars at Kingaroy and Jondaryan (Fig. 3). Cultivar PBA Pistol is a high-yielding cultivar released for Central Queensland, where the frequency of frost events is much smaller due to the warmer growing environment (Chauhan and Ryan 2020). The reasons for high susceptibility in PBA Pistol and PBA Monarch to frost are unknown and require further investigation. This interactions was absent when a combined analysis was performed for all 12 experiments and PBA Pistol, Monarch and Kyabra were not included, suggesting that interactions in the three experiments could be because of differences in the responsiveness of these three additional cultivars.

## Conclusions

This study has provided quantitative evidence that in the southeast Queensland region, which constitutes a significant production area for chickpea, grain yield may be adversely affected by high frequencies of extreme low temperatures, including post-flowering frosts and low-temperature days. Hence, minimising these low temperature-related stresses after flowering will be critical to achieving high grain yields of chickpea in this region. Cultivar susceptibility to frosts could contribute to additional yield losses, though not as large as the growing environment could account for losses in yield. Hence, identifying more tolerant cultivars may also be desirable apart from developing agronomic practices to manage these stresses. Chickpea cultivars should be screened for tolerance to these stresses before recommending them to growers in a region with high frequencies. A screening method should be developed to identify potential cultivars. Cultivars PBA Pistol and PBA Monarch appeared highly susceptible to frosts. These two cultivars could be used as sensitive controls in screening, apart from launching further investigations to understand why they were more susceptible to frost.

## References

[CR1] ABARES (2019) Australian crop report, Australian Bureau of Agricultural and Resources Economics and Sciences, Canberra, September. CC. BY 4.0. 10.25814/5de06ae055ba7

[CR2] Admas S, Haileselassie T, Tesfaye K, Shiferaw E, Flynn KC (2021). Evaluation of Ethiopian chickpea (Cicer arietinum L.) genotypes for frost tolerance. Acta Agric Slov.

[CR3] Anwar MR, Luckett DJ, Chauhan YS, Ip RH, Maphosa L, Simpson M, Warren A, Raman R, Richards MF, Pengilley G (2022) Modelling the effects of cold temperature during the reproductive stage on the yield of chickpea (*Cicer arietinum* L.). Int J Biometeorol 66:111–125 10.1007/s00484-021-02197-8 PMC8727402 34609561

[CR4] Arif A, Parveen N, Waheed MQ, Atif RM, Waqar I, Shah TM (2021). A comparative study for assessing the drought-tolerance of chickpea under varying natural growth environments. Front Plant Sci.

[CR5] Bhandari K, Sharma KD, Hanumantha Rao B, Siddique KH, Gaur P, Agrawal SK, Nair RM, Nayyar H (2017). Temperature sensitivity of food legumes: a physiological insight. Acta Physiol Plant.

[CR6] Chaturvedi S, Mishra D, Vyas P, Mishra N (2009). Breeding for cold tolerance in chickpea. Trends Biosci.

[CR7] Chauhan YS, Ryan M (2020). Frost risk management in chickpea using a modelling approach. Agronomy.

[CR8] Chauhan Y, Allard S, Williams R, Williams B, Mundree S, Chenu K, Rachaputi N (2017). Characterisation of chickpea cropping systems in Australia for major abiotic production constraints. Field Crop Res.

[CR9] Chauhan YS, Ryan M, Chandra S, Sadras VO (2019). Accounting for soil moisture improves prediction of flowering time in chickpea and wheat. Sci Rep.

[CR10] Chauhan Y, Chenu K, Williams R (2021) Using crop modelling to improve chickpea adaptation in variable environments. In: Saxena KB, Saxena RK, Varshney RK (eds) Genetic enhancement in major food legumes. Springer, Cham. 10.1007/978-3-030-64500-7_8

[CR11] Clarke H, Siddique K (2004). Response of chickpea genotypes to low temperature stress during reproductive development. Field Crop Res.

[CR12] Clarke H, Khan TN, Siddique K (2004). Response of chickpea genotypes to low temperature stress during reproductive development. Field Crop Res.

[CR13] Crimp SJ, Zheng B, Khimashia N, Gobbett DL, Chapman S, Howden M, Nicholls N (2016). Recent changes in southern Australian frost occurrence: implications for wheat production risk. Crop Pasture Sci.

[CR14] Croser J, Clarke H, Siddique K, Khan T (2003). Low-temperature stress: implications for chickpea (Cicer arietinum L.) improvement. Crit Rev Plant Sci.

[CR15] Dalal R, Strong W, Weston E, Cooper J, Wildermuth G, Lehane K, King A, Holmes C (1998). Sustaining productivity of a Vertisol at Warra, Queensland, with fertilisers, no-tillage, or legumes. 5. Wheat yields, nitrogen benefits and water-use efficiency of chickpea-wheat rotation. Aust J Exp Agric.

[CR16] Devasirvatham V, Tan DK (2018). Impact of high temperature and drought stresses on chickpea production. Agronomy.

[CR17] Dreccer MF, Fainges J, Whish J, Ogbonnaya FC, Sadras VO (2018). Comparison of sensitive stages of wheat, barley, canola, chickpea and field pea to temperature and water stress across Australia. Agric For Meteorol.

[CR18] Felton W, Marcellos H, Murison R (1996) The effect of row spacing and seeding rate on chickpea yield in northern New South Wales. In: Proceedings of the 8th Australian Agronomy Conference. Toowoomba, Qld, pp 250–253. (The Australian Society of Agronomy)

[CR19] Fischer R (1985). Number of kernels in wheat crops and the influence of solar radiation and temperature. J Agric Sci.

[CR20] Gauch HG (2013). A simple protocol for AMMI analysis of yield trials. Crop Sci.

[CR21] Gowda C, Rao PP, Tripathi S, Gaur P, Deshmukh R (2009) Regional shift in chickpea production in India. In: Ali M, Kumar Shiv (eds) Milestones in food legumes research. Indian Institute of Pulses Research, Kanpur, pp 21–35

[CR22] Heidarvand L, Maali Amiri R, Naghavi M, Farayedi Y, Sadeghzadeh B, Alizadeh K (2011). Physiological and morphological characteristics of chickpea accessions under low temperature stress. Russ J Plant Physiol.

[CR23] Horn C, Birch C, Dalal R, Doughton J (1996). Sowing time and tillage practice affect chickpea yield and nitrogen fixation. 1. Dry matter accumulation and grain yield. Aust J Exp Agric.

[CR24] Jeffrey C, Trethowan R, Kaiser B (2021). Chickpea tolerance to temperature stress: status and opportunity for improvement. J Plant Physiol.

[CR25] Kaloki P, Devasirvatham V, Tan D (2019) Chickpea abiotic stresses: combating drought, heat and cold. Abiotic and biotic stress in plants. Intech Open. 10.5772/intechopen.83404

[CR26] Kirkegaard JA, Lilley JM, Brill RD, Ware AH, Walela CK (2018). The critical period for yield and quality determination in canola (Brassica napus L.). Field Crop Res.

[CR27] Lake L, Sadras VO (2014). The critical period for yield determination in chickpea (Cicer arietinum L.). Field Crop Res.

[CR28] Lake L, Chenu K, Sadras VO (2016). Patterns of water stress and temperature for Australian chickpea production. Crop Pasture Sci.

[CR29] Li Y, Lake L, Chauhan YS, Taylor J, Sadras V (2022) Genetic basis and adaptive implications of temperature-dependent and temperature-independent effects of drought on chickpea phenology. J Exp Bot 73(14): 4981–4995. 10.1093/jxb/erac195 10.1093/jxb/erac195 35526198

[CR30] Lilley JM, Bell LW, Kirkegaard JA (2015). Optimising grain yield and grazing potential of crops across Australia’s high-rainfall zone: a simulation analysis. 2. Canola. Crop Pasture Sci.

[CR31] Longin CFH, Sieber AN, Reif JC (2013). Combining frost tolerance, high grain yield and good pasta quality in durum wheat. Plant Breed.

[CR32] Maqbool A, Shafiq S, Lake L (2010). Radiant frost tolerance in pulse crops—a review. Euphytica.

[CR33] Muehlbauer FJ, Sarker A (2017) Economic importance of chickpea: production, value, and world trade. In: Varshney R, Thudi M, Muehlbauer F (eds) The chickpea genome. Compendium of plant genomes. Springer, Cham. 10.1007/978-3-319-66117-9_2

[CR34] Oweis T, Hachum A, Pala M (2004). Water use efficiency of winter-sown chickpea under supplemental irrigation in a Mediterranean environment. Agric Water Manag.

[CR35] Pouresmael M, AD S, Kanouni H, Bokaei A, Mahdieh M (2020) Screening of desi type chickpea accessions collection of the National Plant Gene Bank of Iran for cold tolerance under field conditions. Seed and Plant 36(3):377–382. Retrieved from https://sid.ir/en/Journal/ViewPaper.aspx?ID=822564

[CR36] Pyett S, de Vet E, Trindade L, van Zanten H, Fresco L (2019) Chickpeas, crickets and chlorella: our future proteins. Wageningen Food & Biobased Research, Wageningen. Retrieved from https://edepot.wur.nl/496402

[CR37] Rachaputi RC, Sands D, McKenzie K, Chauhan Y, Bell K, Seyoum S, Agius P, Krosch S, Lehane J (2021). Can partial reduction of shoot biomass during early vegetative phase of chickpea save subsoil water for reproductive and pod filling?. Agric Water Manag.

[CR38] Raiya R, Hegde V, Krishnan V, Bharadwaj C, Tripathi S, Jain PK (2021). Genetics of lodging resistance in chickpea (Cicer arietinum L). Euphytica.

[CR39] Sadras V, Dreccer MF (2015). Adaptation of wheat, barley, canola, field pea and chickpea to the thermal environments of Australia. Crop Pasture Sci.

[CR40] Sandaña P, Calderini DF (2012). Comparative assessment of the critical period for grain yield determination of narrow-leafed lupin and pea. Eur J Agron.

[CR41] Singh G, Ram H, Aggarwal N, Turner NC (2016). Irrigation of chickpea (Cicer arietinum L.) increases yield but not water productivity. Exp Agric.

[CR42] Soureshjani HK, Dehkordi AG, Bahador M (2019). Temperature effect on yield of winter and spring irrigated crops. Agric for Meteorol.

[CR43] Stagnari F, Maggio A, Galieni A, Pisante M (2017). Multiple benefits of legumes for agriculture sustainability: an overview. Chem Biol Technol Agric.

[CR44] Takashima NE, Rondanini DP, Puhl LE, Miralles DJ (2013). Environmental factors affecting yield variability in spring and winter rapeseed genotypes cultivated in the southeastern Argentine Pampas. Eur J Agron.

[CR45] Upadhyaya HD, Dronavalli N, Gowda C, Singh S (2011). Identification and evaluation of chickpea germplasm for tolerance to heat stress. Crop Sci.

[CR46] Wang B, Chen C, Li Liu D, Asseng S, Yu Q, Yang X (2015). Effects of climate trends and variability on wheat yield variability in eastern Australia. Climate Res.

[CR47] Wery J, Turc O, Lecoeur J (1993) Mechanisms of resistance to cold, heat and drought in cool-season legumes, with special reference to chickpea and pea. In: Singh K, Saxena M (eds) Breeding for stress tolerance in cool-season food legumes. Wiley & Sons, Wiley, Chichester, pp 271–291

[CR48] Whish J, Castor P, Carberry P, Peake A (2007). On-farm assessment of constraints to chickpea (Cicer arietinum) production in marginal areas of northern Australia. Exp Agric.

[CR49] Zaman-Allah M, Jenkinson DM, Vadez V (2011). A conservative pattern of water use, rather than deep or profuse rooting, is critical for the terminal drought tolerance of chickpea. J Exp Bot.

[CR50] Zheng B, Chapman SC, Christopher JT, Frederiks TM, Chenu K (2015). Frost trends and their estimated impact on yield in the Australian wheatbelt. J Exp Bot.

